# Raman Spectroscopy for Monitoring NO_x_ and N_2_O in Combustion Products

**DOI:** 10.3390/s26103180

**Published:** 2026-05-17

**Authors:** Riccardo Dal Moro, Fabio Melison, Lorenzo Cocola, Luca Poletto

**Affiliations:** National Research Council of Italy, Institute for Photonics and Nanotechnologies, CNR-IFN, Via Trasea 7, 35131 Padova, Italy; fabio.melison@cnr.it (F.M.); lorenzo.cocola@cnr.it (L.C.); luca.poletto@cnr.it (L.P.)

**Keywords:** Raman spectroscopy, combustion, gas sensing, NO_x_, NO_2_, combustion diagnostic

## Abstract

The increasing adoption of alternative fuels such as hydrogen and ammonia in energy systems has created a growing need for advanced diagnostic techniques capable of monitoring combustion products with high specificity and flexibility. In this context, Raman spectroscopy represents a promising optical approach for gas analysis, as it enables the simultaneous detection of multiple species without requiring sample preparation. In this work, the performance of a cost-effective Raman-based system on quantitative detection of nitrogen oxides (NO and NO_2_) and nitrous oxide (N_2_O) is presented. The experimental setup is based on a multi-pass optical configuration designed to enhance the Raman signal and employs off-the-shelf components, including an uncooled CMOS detector. Calibration measurements were carried out using gas mixtures at known partial pressures, and gas concentrations were retrieved through a nonlinear least-squares fitting procedure applied to the measured spectra. The results show that the system provides linear and repeatable responses for NO and N_2_O over the investigated pressure ranges, with low mean errors and limited data dispersion, while NO_2_ performance could not be fully quantified in a comparable manner due to the high reactivity of the species under the tested conditions. Overall, the proposed system represents a viable and cost-effective solution for multi-species gas analysis in emerging combustion applications. This work aims to extend the industrial applicability of Raman spectroscopy to NO_x_ and NO_2_ diagnostics.

## 1. Introduction

After years of relying on conventional combustion processes, collective efforts are now shifting toward the decarbonization of energy production systems. Carbon dioxide is one of the main Green House Gases (GHGs), and its anthropogenic component is continuously increasing due to the constant rise in energy demand. Total energy-related CO_2_ emissions increased by 0.8% in 2024, hitting an all-time high of 37.8 Gt CO_2_ [1].

New forms of non-traditional combustion, such as hydrogen [2,3,4] and ammonia combustion [5], are gaining appeal. Hydrogen combustion is very clean under ideal conditions, producing only water molecules as by-products. However from an application standpoint, its practical use is limited by the complex requirements for liquid-phase storage, which reduces its attractiveness in terms of the energy-to-cost ratio, constraining its widespread deployment in conventional energy systems [2,6]. Ammonia, on the other hand, can be used as a hydrogen carrier and can be stored in liquid form more easily than hydrogen. As interest in this fuel grows, it becomes essential to develop new diagnostic tools for combustion processes that are cost-effective and can be directly implemented in-line within energy production systems.

Among the main combustion products of pure ammonia, carbon-based compounds are absent; however, other potentially problematic species, such as nitrogen oxides (NO_x_) and nitrous oxide (N_2_O), can be formed [7,8,9]. Nitrogen oxides are among the most critical pollutants generated during combustion processes and must be monitored due to their significant environmental and health impacts [10]. NO_x_ species, primarily nitric oxide (NO) and nitrogen dioxide (NO_2_), play a key role in the formation of ground-level ozone and photochemical smog which degrade air quality and harm vegetation. They also contribute to acid rain, leading to soil and water acidification and damaging ecosystems. From a health perspective, exposure to NO_x_ can irritate the respiratory system, exacerbate asthma and increase vulnerability to infections [11,12]. Additionally, nitrous oxide is a potent greenhouse gas with a Global Warming Potential (GWP) about 300 times higher than that of CO_2_ and 25 times that of the methane (CH_4_) [8,13]. So, even the formation of a limited amount of N_2_O during ammonia combustion can offset the advantage provided by the absence of CO_2_ emissions in terms of GWP.

The combined environmental and health effects of NO_x_ and N_2_O emissions make their continuous monitoring a crucial aspect in the development of sustainable and low-emission new combustion technologies.

For combustion applications, ammonia is often blended with other gaseous fuels [8,14,15], such as methane or hydrogen, because its standalone combustion is characterized by low flame speeds, narrow flammability limits and the tendency to produce significant fuel-NO_x_ emissions. Blending with a more reactive fuel improves ignition characteristics, enhances flame stability, and extends the operational range, allowing robust operation even in systems originally designed for hydrocarbon fuels. Experimental investigations further show that NH_3_/CH_4_ mixtures widen the stable combustion window and improve process stability compared to pure ammonia, although they may also lead to increased NO_x_ emissions depending on operating conditions [15,16]. Because co-combustion activates multiple chemical pathways it is essential to monitor all species formed during oxidation. According to the kinetic modeling by Pedersen et al. [8], NO_x_ and N_2_O concentrations vary significantly depending on the equivalence ratio (ϕ) and temperature. In pure ammonia combustion, N_2_O concentration can reach 5000 ppm during the ignition phase, while NO and total NO_x_ levels can exceed 8000 ppm and 10,000 ppm, respectively (e.g., at high temperatures, as shown in Figure 5e of [8]). Comprehensive speciation, including NO_x_, unburned NH_3_, CO, H_2_ and intermediate nitrogenous compounds, is necessary to ensure accurate characterization of combustion behavior and compliance with environmental and safety requirements.

Combustion gas diagnostics are commonly performed using laboratory-based analytical systems such as gas chromatography (GC) or mass spectrometry (MS) [17,18]. These systems provide excellent detection performance but require supervision by highly specialized personnel and involve considerable operational costs. Moreover, they can be sensitive to environmental conditions and typically require specific sample preparation. For these reasons, their in-line direct implementation is not straightforward, and they are unable to provide data with a sufficiently high sampling rate to capture the dynamic behavior of the combustion process. In industrial settings, a widely adopted alternative is the use of electrochemical sensors [19,20]. This solution is particularly advantageous from the economic side; however, it may present challenges related to the effective interaction between the gas flow and the sensing element. The major issues with electrochemical gas sensors are associated with cross-sensitivity to interfering species and hysteresis effects occurring during their operation. Additionally, these devices generally have a relatively short lifetime and require periodic maintenance to ensure proper operation.

Optical diagnostic techniques provide a robust alternative to the aforementioned methods, enabling time-resolved characterization of chemical species in combustion gases without the need for specific sample preparation. In addition, their primary advantage lies in the fact that no direct contact is required between the gas mixture under analysis and the transducer element of the system; this significantly enhances their benefits when compared to contact-based transducers. The main optical gas analysis techniques are infrared (IR) absorption spectroscopy methods, such as Fourier Transform Infrared Spectroscopy (FT-IR) [21,22] and Tunable Diode Laser Absorption Spectroscopy (TDLAS) [23,24], as well as Laser-Induced Fluorescence (LIF) [25,26,27] techniques, chemiluminescence methods [28] and Raman spectroscopy-based approaches [29,30,31,32].

Infrared absorption spectroscopy techniques are based on the principle that target molecules absorb radiation at different energy levels. Specifically, the FT-IR approach enables the analysis of a broad absorption spectrum, providing information on multiple chemical species simultaneously. In contrast, the TDLAS technique focuses on individual absorption features of specific molecules and requires a dedicated measurement channel (laser source and detector) for each target molecule to be measured. A drawback of analysis techniques based on this principle is their limited ability to detect diatomic molecules such as H_2_ [33,34] and N_2_. This limitation makes IR absorption techniques less suitable for applications involving the use of H_2_ as a fuel. Fluorescence and chemiluminescence analysis techniques allow accurate measurement of NO_x_ concentrations but provide information only for a restricted set of molecular species, thus requiring integration with additional measurement systems to obtain a comprehensive characterization of the whole combustion products.

An optical technique capable of simultaneously detecting species such as H_2_, N_2_, O_2_, as well as numerous other molecules, including NO_x_, is Raman spectroscopy [35,36]. This spectroscopic method is based on the phenomenon of Raman scattering: when light interacts with molecules, photons can be scattered inelastically. Thereby, these inelastic-scattered photons exhibit a difference in energy compared to that of the exciting radiation. This difference in energy is referred to as the Raman shift (ν) and provides information about the roto-vibrational states of the gaseous molecules and their chemical bonds, thereby enabling the identification of the chemical species present in the sample under analysis. The quantity of photons exhibiting a Raman shift is directly proportional to the power of the incident light and the inverse of the fourth power of the excitation photon wavelength. It also depends on the intrinsic properties of the molecules (cross-section) and the density of the molecules in the light-matter interaction volume. Analysis using this spectroscopic technique can be conveniently performed by using a monochromatic laser source centered in the visible spectrum, thus avoiding the necessity for infrared optics and detectors. While this type of analysis is extensively used for characterizing solid and liquid samples, the low density of gaseous samples presents a significant challenge that must be addressed during instrument design [29,37,38]. Although spontaneous Raman scattering applied to gas analysis is not yet a widespread technique in industrial settings, recent studies continue to demonstrate its broad applicability in various scientific and industrial contexts: key contributions to the application of Raman spectroscopy for gas analysis include the work of Eckbreth [32] in the field of combustion diagnostics and Schrötter [35] for more general applications. Significant studies have been reported for example by the research groups of Petrov [39,40,41], Seeger [31,38,42], and Poletto [29,43,44,45].

To extend the application of gas Raman spectroscopy to non-traditional combustion products, this work presents specific results on the detection performance of NO_x_ and NO_2_. The analysis utilizes an instrument [29] designed for Raman signal enhancement, which was characterized by a broad range of species in conventional combustion, including N_2_, O_2_, CO_2_, H_2_, CH_4_, CO, SO_2_, H_2_S, and H_2_O.

## 2. Materials and Methods

### 2.1. Experimental Setup

**Figure 1:**
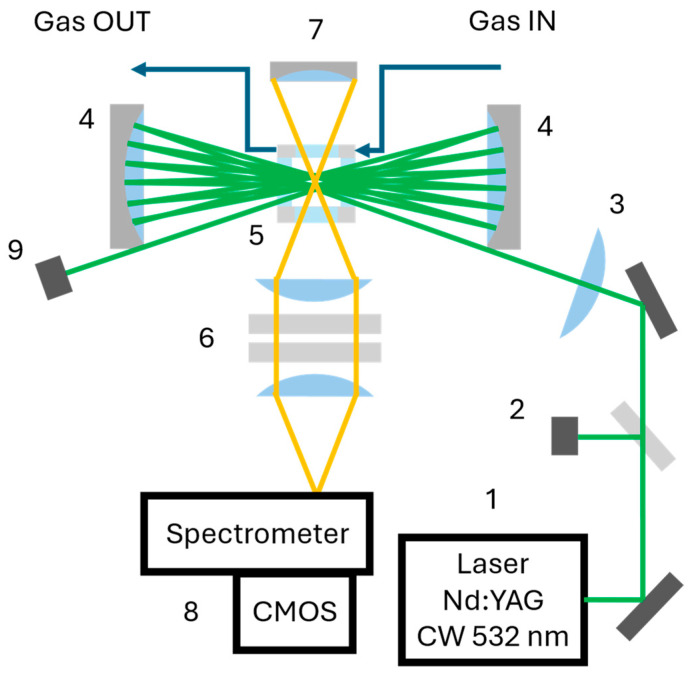
Schematic representation of the instrument setup with its main components highlighted: 1. Laser source; 2. Photodiode; 3. Focalizing lens; 4. Spherical mirrors; 5. Gas analysis cell; 6. Collection optics; 7. Back-reflection mirror; 8. Spectrometer; 9. Beam dump.

The experimental apparatus is based on the analysis of spontaneous Stokes Raman scattering generated within a compact, windowed gas cell. A schematic representation of the setup is shown in Figure 1. The Raman-scattered radiation is collected at 90° with respect to the excitation beam by a lens-based optical system and directed toward a custom-made diffraction grating spectrometer.

The excitation source is a continuous-wave Neodymium-Doped Yttrium Aluminum Garnet (Nd:YAG) laser (CNI Laser, Changchun, China) (1) operating at 532 nm with an optical power of 1 W and a spectral width of 0.3 nm. The laser beam is focused into the gas cell (5) by means of a 200 mm focal length lens (3). To enhance the Raman signal intensity, a multi-pass optical configuration is implemented. Two spherical mirrors (4) with a focal length of 50 mm redirect the beam through the interaction volume, producing a total of 19 passes across the focal region. The choice of the number of reflections was governed by a compromise between system dimensions and alignment tolerances; this configuration significantly increases the effective photon density within the sample without requiring higher laser power. To increase the photons collected by the spectrometer (8) a spherical back-reflection mirror (7) positioned opposite to the collection optics reflects backward-emitted radiation into the detection path, thereby increasing the collected Raman signal: by analyzing the signal associated with the N_2_ vibrational band (at 2331 cm^−1^) in ambient air, an increase in signal by a factor of 1.85 is observed compared to the configuration without the mirror. After passing through the gas cell, the laser beam is safely terminated in a beam dump (9). A normalization photodiode (2) (OPT101, Texas Instrument, Dallas, TX, USA) is positioned upstream of the gas cell, where a small fraction of the laser beam is diverted and subsequently monitored. A digital pressure gauge (Druck DPI 104, Leicester, UK) is connected to the gas cell. Its readings, with an accuracy of 0.1 mbar, are used to determine the actual internal total pressure in order to correlate the partial pressures of the species under analysis.

In order to prevent corrosion caused by aggressive molecules, the gas cell is fabricated from stainless steel using CNC machining and is designed to minimize its internal volume while allowing operation both above and below ambient pressure. Optical access is provided by 40 mm anti-reflection V-Coat λ/4 N-BK7 windows optimized for 532 nm radiation (Edmund Optics, Barrington, NJ, USA) on the laser line side and 25 mm broadband visible coated λ/4 N-BK7 windows (Edmund Optics) on the collection and back-reflection mirror sides. Prior to entering the cell, gas samples pass through particulate and oil filters to prevent contamination of the optical components. This is the only sample preparation performed prior to injection into the system.

The Raman signal is analyzed using a custom-built spectrometer based on a f/2.4 photographic-lenses design and a 1200 grooves/mm diffraction grating (Edmund Optics). Detection is performed by an industrial-grade CMOS monochromatic camera (Basler ACE 1920–40 μm, Ahrensburg, Germany) equipped with an uncooled detector (Sony IMX249, 1920 × 1200 pixels, 5.86 μm pixel size, Tokyo, Japan). All optical and mechanical components are assembled inside a transportable enclosure with dimensions of 750 × 450 × 440 mm. All analyses and results presented in this work were conducted under standard laboratory ambient conditions (20–25 °C), with the internal detector temperature stabilizing between 47 and 52 °C. Both the CMOS sensor and the laser source require a warm-up period of approximately 5 min to ensure measurement repeatability. Regarding the thermal robustness of the system, it should be noted that while the CMOS sensor maintains satisfactory performance over a wide range of temperatures, the overall operating range of the instrument is currently limited by the laser source (10–35 °C). Within this specific window, the system’s performance remains stable without the need for active thermal management. However, operation in environments exceeding these limits would necessitate an active temperature control system to ensure the stability of the laser emission and the integrity of the spectral acquisition.

To reduce data volume and processing time, a 4 × 4 pixel binning is applied, resulting in effective frames of 480 × 300 pixels and an average spectral resolution of 7.3 cm^−1^ per pixel. The accessible Raman shift range extends from 587 cm^−1^ (H_2_ rotational band) to 3657 cm^−1^ (H_2_O vibrational band). By rotating the diffraction grating, the detection window can be shifted to include the H_2_ vibrational band at 4156 cm^−1^.

The scattered photons are collected and relayed by a two-stage optical system. Collimation is achieved using an Hastings achromatic triplet with a focal length of 40 mm (Thorlabs, Newton, NJ, USA), while a second Hastings achromatic triplet with a focal length of 20 mm (Thorlabs) focuses the beam onto the entrance slit of the spectrometer. The ratio between the focal lengths of the two lenses results in an overall imaging demagnification factor of 2. The adjustable entrance slit is used to balance spectral resolution and stray light suppression with an opening of 550 μm that was found to provide optimal performance. A long-pass filter (Thorlabs) with a cut-off wavelength of 550 nm is placed in a collimated section of the optical path to remove the strong Rayleigh component of the scattered light.

Laser safety is ensured by enclosing the excitation beam within protective panels, fully complying with standard safety requirements. The system is designed with emphasis on robustness, portability, and cost-effectiveness, relying primarily on commercially available components.

### 2.2. Spectrum Generation

The spectrum generation is based on the imaging analysis of the Raman photons scattered by the sample and decomposed into its spectral components by the diffraction grating. As stated in the previous section, the detector has a resolution of 1920 × 1200 pixels that are reduced to 480 × 300 pixels via a 4× binning operation.

**Figure 2:**
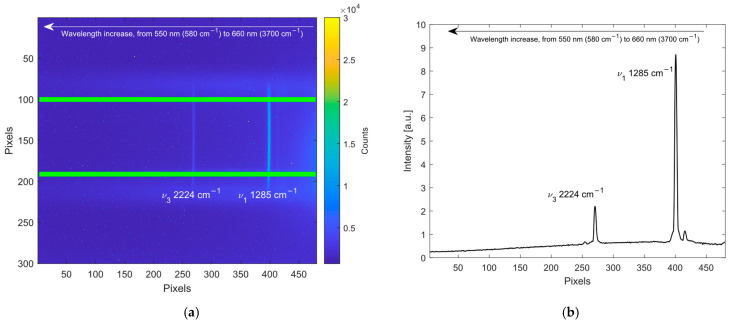
Example of spectrum generation: (**a**) frame acquired during the analysis of a mixture of N_2_O and Argon, ROI highlighted in green; (**b**) spectrum computed from the reported frame. The N_2_O features ν_1_ at 1285 cm^−1^ and ν_3_ at 2224 cm^−1^ are highlighted in both panels.

Figure 2a shows, as an example, a frame acquired with 10 s integration time during the analysis of a mixture composed of N_2_O and Argon (non-active Raman molecule). The vertical bands represent the diffracted image of the laser beam (the N_2_O features ν_1_ at 1285 cm^−1^ and ν_3_ at 2224 cm^−1^ [35,46] are highlighted) with the wavelength increasing from the right to the left. The boundaries of the region of interest (ROI), used to compute the spectrum, are highlighted in green. The spectrum related to the frame is reported in Figure 2b; it is obtained by summing the intensities of the pixels for each column in the ROI. Before this operation, the frame is compensated by the dark frame and then is elaborated in order to correct curvature effects: ideally the spectral lines should be straight but the optical aberrations curve their image onto the CMOS sensor. If curvature is not corrected, the spectrum broadens, leading to a loss of sensitivity and resolution. Finally, the spectra are normalized by the photodiode signal to compensate for any variation in the optical power of the laser source.

Since all spectra are computed in the detector reference frame (pixel number), conversion from the pixel scale to the wavenumber (cm^−1^) scale is required to express the data in spectral units. To perform this coordinate change, the spectra x-axis is correlated using a second-order polynomial interpolation that relates the pixel’s position with reference positions of known Raman features [35] (e.g., H_2_O at 3657 cm^−1^, N_2_ at 2231 cm^−1^, CO_2_ at 1388 cm^−1^ and 1285 cm^−1^, O_2_ at 1555 cm^−1^ and CH_4_ at 2914 cm^−1^). This pixel-to-wavenumber conversion is primarily intended for spectral visualization and physical interpretation, as the analysis algorithm operates directly in the pixel domain.

### 2.3. Methods for Spectral Analysis

The quantitative results are expressed in terms of the partial pressure of the target molecule present in the mixture. Quantification is performed indirectly by comparing the measured spectrum with calibration spectra acquired at known partial pressures. The partial-pressure information is retrieved through a fitting technique based on a nonlinear least-squares approach, where the minimization of the residuals is performed using the cost function described in Equation (1):

(1) $$\varepsilon \left(n\right)=sp_{synth}(n)-sp_{meas}(n)$$

where $\varepsilon \left(n\right)$ represents the difference at the “*n*” pixel between the computed synthetic ($sp_{synth}$) and the measured ($sp_{meas}$) spectrum. The synthetic spectrum is computed as shown in Equation (2):


(2) $$sp_{synth}=\left(\sum_{i=1}^{n}\alpha_{i}\cdot Calib_{i}\left(n\right)\right)+a+b\cdot n+c\cdot n^{2}$$

Again, “n” represents the pixel under analysis; it ranges from 1 to N where N is the pixel-length of the spectral window used in the fitting process. The term $\alpha_{i}$ is a multiplication factor for the calibration spectrum of the i-molecule at the n-pixel (defined in Equation (2) as $Calib_{i}\left(n\right)$). A second-order polynomial is used to compensate for the background light affecting the spectrum in the window. This background is a cumulative contribution of ambient stray light, fluorescence originating from the optical windows, intrinsic fluorescence of the molecules under analysis and fluorescence emitted by potential contaminants within the gas mixture. The analysis of NO_x_ species is complicated by fluorescence interference, particularly from NO_2_, which emits across the spectrum toward longer wavelengths. As long as the fluorescence signal does not saturate the detector, it is still possible to extract quantitative information from the Raman features: the saturation threshold for NO_2_ at an integration time of 10 s is 10 mbar (1% *v*/*v* at atmospheric pressure). Above this partial pressure, it is necessary to reduce the integration time to prevent detector saturation.

The value of the measured partial pressure (*PP_m_*) is obtained by the multiplication of the calibration partial pressure (*PP_calib_*) and the $\alpha_{i}$ factor as shown in Equation (3).


(3) $$PP_{m}=\alpha_{i}\times PP_{calib}$$

**Figure 3:**
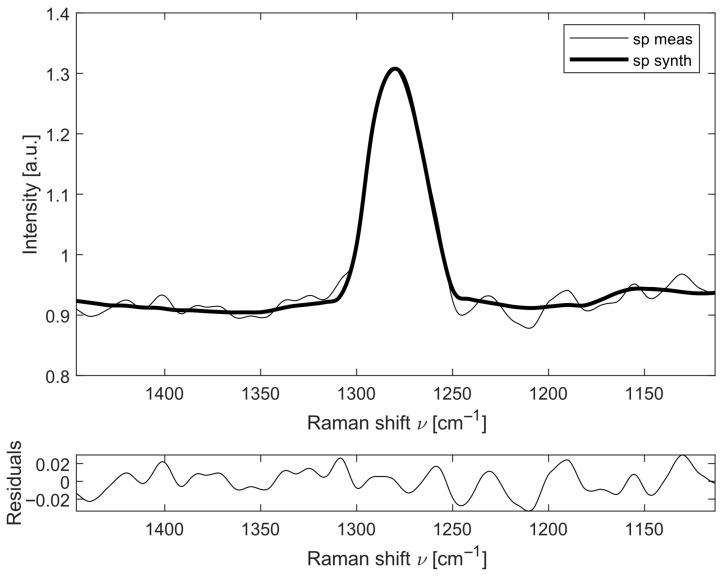
Example of the residuals between the synthetic spectrum (thick line) compared to the measured spectrum (thin line) of N_2_O at 13 mbar of partial pressure.

The calibration spectra for gas quantification were obtained by averaging 30 spectra acquired at known partial pressures. An example of the fitting result is shown in Figure 3: the measured N_2_O spectrum at a partial pressure of 13 mbar (thin line), the computed synthetic spectrum (thick line), and the residuals between the two, in a region centered at 1280 cm^−1^. The quality of the fitting procedure is assessed by analyzing the residuals between the synthetic and measured spectra: any systematic patterns in these residuals indicate whether a species is incorrectly fitted or an unmodeled spectral feature is present. As shown in Figure 3, the fitting algorithm accurately reproduces the spectral peaks, and the magnitude of the residuals is two orders of magnitude lower than that of the analyzed signal. The spectral windows within which the analysis was performed were selected to avoid spectral overlap among the three molecules, while identifying regions of strong features for each of them. The dominant species in air-breathing combustion, such as N_2_ (2331 cm^−1^) and O_2_ (1555 cm^−1^), exhibit Raman shifts well-separated from those of NO (1877 cm^−1^), NO_2_ (750 cm^−1^) and N_2_O (1280 cm^−1^), as well as other typical components like NH_3_ (3334 cm^−1^), H_2_ (4156 cm^−1^) and H_2_O (3657 cm^−1^). Consequently, their presence does not compromise the detection accuracy for lower-concentration species.

The influence of pressure-induced broadening on the Raman lines was found to be negligible within the operational range of the system. In this setup, any potential broadening effect related to pressure variation is dominated and effectively masked by the larger spectral linewidth of the excitation laser source and the resolution of the spectrometer. Consequently, the spectral profile is primarily dominated by the laser’s bandwidth and the spectrometer’s resolution. Thus, the quantitative retrieval of gas concentrations remains robust against pressure-induced spectral distortions.

### 2.4. Test Routine

The tests were performed at ambient temperature by measuring different partial pressure levels of the target gas under static conditions inside the cell, using a calibrated gas mixture. The signal integration time was set to 10 s per acquired spectrum to simulate steady-state combustion conditions. Limits of Detection (LODs) were computed as reported in previous work of the authors [29].

## 3. Results

### 3.1. Nitrogen Oxide (NO)

**Figure 4:**
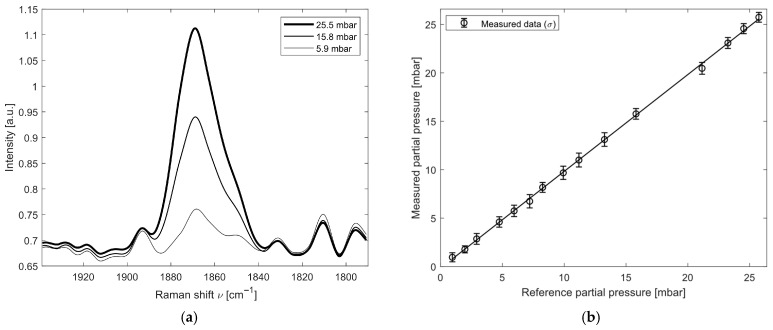
Results for the NO spectral analysis: (**a**) Raman feature of NO at 1877 cm^−1^ at different partial pressures; (**b**) measured partial pressure of NO and its standard deviation σ.

Regarding the analysis of NO, the detection limit of the instrument (using a spectral integration time of 10 s) is 1 mbar. Starting from a certified gas cylinder (RISAM GAS) containing 0.4% *v*/*v* of NO in 99.6% *v*/*v* N_2_, partial pressures of NO were analyzed over a range from 1 to 25 mbar. For the calibration and subsequent analysis, the spectral feature around the Raman spectral band at 1877 cm^−1^ was used. Figure 4a shows the spectral band used for instrument calibration at 25, 16 and 6 mbar of NO. The results of the linearity test are reported in Figure 4b and Table 1. For each partial-pressure step, 30 measurements were acquired, and the mean measured value with its corresponding standard deviation is shown. The measurement bias was calculated as the difference between the reference partial pressure and the measured partial pressure provided by the instrument as the output of the spectral analysis through the fitting routine.

As shown in Table 1, the signal response provided by the instrument is linear and repeatable across the entire measurement range under investigation. The average measured bias is −0.16 ± 0.14 mbar.

### 3.2. Nitrous Oxide (N_2_O)

**Figure 5:**
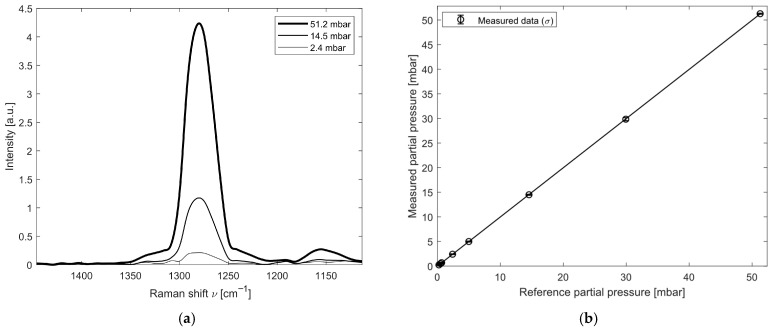
Results for the N_2_O spectral analysis: (**a**) Raman feature of N_2_O at 1280 cm^−1^ at different partial pressures; (**b**) measured partial pressure of N_2_O and its variation σ.

The linearity analysis for N_2_O was carried out using pure gas (purity > 99.9%) diluted with a Raman-inactive gas (Argon) for a concentration of N_2_O of 4.9505%. Partial pressures of N_2_O ranging from 0.2 to 50 mbar were analyzed and the detection limit of this molecule is 0.2 mbar. For the analysis of this molecule, the spectral window around 1280 cm^−1^ was investigated. Figure 5a shows the spectral feature measured at a partial pressure of 51.2 mbar of N_2_O, as well as at partial pressures of 14.5 and 2.4 mbar. The results of the linearity test are reported in Figure 5b and Table 2; 30 measurements were acquired for each partial-pressure step.

For this molecule as well, the signal response provided by the instrument is linear and repeatable across the entire measurement range. As reported in Table 2, the measurements exhibit an average error of −0.016 ± 0.04 mbar.

### 3.3. Nitrogen Dioxide (NO_2_)

Since nitrogen dioxide is reactive and interacts with the residual moisture content in the pipes and filters [47,48], it was not possible to perform the linearity test as done for the other molecules. Instead of conducting a linearity analysis of the measurement, an analysis was carried out on the residence trend of the molecule in the cell over time. The gas mixture analyzed consisted of 0.1% *v*/*v* of NO_2_, 79% *v*/*v* N_2_ and 20.9% *v*/*v* O_2_ (RISAM GAS), and the cell was filled with 1.1 bar of the mixture.

**Figure 6:**
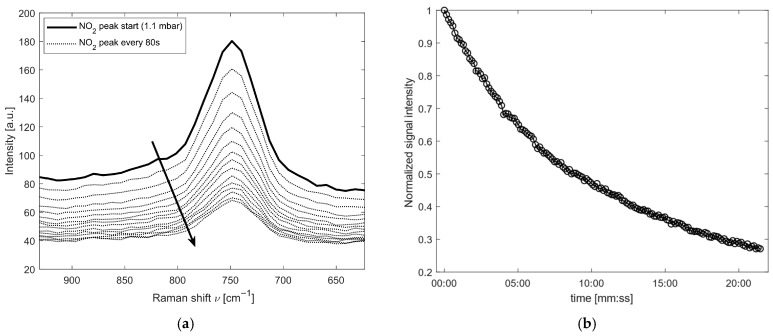
Results for the NO_2_ spectral analysis: (**a**) Raman feature of NO_2_ at 750 cm^−1^ over time, starting from 1.1 mbar. The arrow indicates the time evolution during the acquisition; (**b**) evolution of the signal of the NO_2_ during the test.

By analyzing the stability of the gas inside the cell and using the fit output as the reference value (calibrated on the spectral feature at a Raman shift of 750 cm^−1^), the behavior of NO_2_ over time was evaluated. Figure 6a shows the corresponding spectra measured during the acquisition cycle; the arrow indicates the time evolution during the acquisition. As can be observed, in addition to a variation in the peak intensity, there is also a significant decrease in the background signal, which is attributed to fluorescence generated by the interaction with the 532 nm excitation light. Figure 6b reports the temporal evolution of the measured NO_2_ signal. A progressive decrease is evident, with a reduction of approximately 70% over 20 min. Considering that the initial partial pressure is 1.10 mbar, the nitrogen dioxide reacts over time, reaching 0.30 mbar at the end of the test.

## 4. Discussion

To contextualize the performance of the proposed Raman system, its analytical capabilities were compared with the typical emission profiles of ammonia-fueled combustion. Based on kinetic modeling [8], species such as NO_x_ and N_2_O exhibit extreme concentration dynamics, with peaks reaching 5000 ppm for N_2_O and exceeding 10,000 ppm for total NO_x_ during critical phases. In this context, the experimental LODs of the proposed instrument, 1 mbar for NO and 0.2 mbar for N_2_O, corresponding to 1000 ppm and 200 ppm at atmospheric pressure, respectively, are well suited for monitoring these species under high-concentration conditions without risk of sensor saturation.

As summarized in Table 3, while other established diagnostic techniques offer specific advantages, they often struggle with the unique demands of ammonia combustion monitoring. Electrochemical sensors, despite their low cost and ease of installation, are limited by a narrow operational range and suffer from possible cross-sensitivity and limited lifespan when exposed to harsh exhaust environments. Conversely, FTIR and LIF provide excellent sensitivity and lower LODs but their industrial application is hampered by high costs, bulky setups, and complex calibration requirements. Unlike multi-gas spectroscopic techniques, GC typically requires coupling with other analyzers to provide a comprehensive characterization of the mixture, as a single-column setup often cannot simultaneously resolve all species of interest. Furthermore, a distinct advantage of our system is the capability to perform ‘wet’ gas analysis directly: unlike many infrared-based techniques or GC analysis where water vapor causes significant spectral overlap or column degradation, the Raman signal from water vapor does not interfere with those of other target molecules, eliminating the need for water removal systems.

Currently, no commercial Raman analyzers for multi-species gas analysis in combustion environments are available on the market, as most of the literature on Raman gas spectroscopy focuses on high-end laboratory setups. Our work aims to bridge the gap between fundamental research and real-world industrial applications by leveraging a cost-effective design. This architecture is centered on an uncooled CMOS detector, off-the-shelf optical components selected for a low f/# spectrometer, and a CW laser characterized by a relatively broad spectral width.

Compared to high-end laboratory Raman setups recently reported in the literature [40,46] our system offers a substantial hardware simplification. While typical laboratory-grade configurations often rely on high-cost components—such as cooled CCDs, high-power lasers, high-pressure samples or long integration times—our architecture achieves reliable quantification using more accessible components and significantly shorter signal integration periods. This reduced integration time represents a key operational advantage, providing the temporal resolution required for in-line monitoring and process control in energy production systems.

The proposed Raman-based approach, in its current configuration, is not intended for trace gas detection where sub-ppm or ppb sensitivity is required. Such levels of sensitivity are typically the domain of more complex and expensive techniques, such as LIF or Chemiluminescence Detection (CLD) [53]. However, for a vast range of industrial and energy-related applications where the species of interest are typically present in concentrations exceeding 500 ppm, this system demonstrates significant potential. In these scenarios, the trade-off between extreme sensitivity and the benefits of robustness, cost-efficiency, and simultaneous multi-species detection makes the Raman-based analyzer a highly practical and viable diagnostic tool for industrial environments.

## 5. Conclusions

The performance of the proposed Raman-based prototype for the detection of combustion-related species, such as nitrogen oxides (NO and NO_2_) and nitrous oxide (N_2_O), has been experimentally evaluated. The system demonstrated the capability to provide quantitative results in NO and N_2_O detection over the investigated range of partial pressures for the target molecules. The adopted spectral fitting approach, based on calibration datasets acquired at known partial pressures and a nonlinear least-squares procedure, proved effective in extracting reliable quantitative information from the measured spectra.

The instrument response was found to be linear and repeatable for NO and N_2_O, with low mean bias and limited data dispersion, confirming the validity of the calibration methodology. In contrast, NO_2_ exhibited a different behavior due to its reactive nature, leading to a progressive decrease in signal intensity over time under static conditions.

The measurable range of the instrument can be extended to higher partial pressures by adjusting the signal integration time, allowing the system to operate over a broader range of partial pressures than those investigated in this work. This flexibility represents a key advantage for adapting the measurement conditions to different applications. The partial pressures studied in this refer to practical conditions measurable in ammonia combustion processes.

A major strength of the proposed approach lies in its capability to detect multiple gas species simultaneously within combustion mixtures while maintaining a relatively low system cost. This is achieved through the use of commercial off-the-shelf components and a compact optical design. The use of an uncooled detector contributes to the overall cost-effectiveness of the system, although it introduces higher noise levels compared to cooled devices. From a technological perspective, further improvements in sensitivity and detection limits could be achieved by adopting a higher-performance cooled detector.

The Raman system proposed here bridges the gap between low-cost sensors and laboratory-grade instrumentation. It enables in situ measurements of ‘wet’ combustion gas with a sampling rate (0.1 Hz) that is significantly faster than GC techniques and sufficient for monitoring the chemical dynamics of NH_3_ steady state combustion. By providing simultaneous multi-gas detection in a single, robust, and low-maintenance setup, this system demonstrates high industrial viability, offering a scalable alternative for process control in carbon-free energy systems where traditional sensors would face saturation or chemical degradation.

### Future Development

To further enhance the accuracy and stability of NO_2_ measurements, future iterations of the system will focus on mitigating the high reactivity and adsorption tendencies of this species. While the current study was conducted under static conditions due to the limited volume of the available high-accuracy certified calibration cylinders, transitioning to a continuous flow-through configuration is expected to reduce surface-catalyzed degradation. Additionally, a controlled heating system for both the gas inlet lines and the Raman cell will be implemented to minimize NO_2_ interaction with residual moisture.

Future developments will focus on the miniaturization of the prototype and on extending its application to in-line analysis of combustion processes, including biogas systems and alternative fuels such as ammonia and hydrogen. Wiener estimation [54] and other advanced denoising algorithms will be evaluated in future works to further push the analytical limits of the instrument.

## Figures and Tables

**Table 1:** Results of the test for the nitrogen oxide detection.

Total Pressure [mbar]	Partial Pressure [mbar]	Measured Partial Pressure [mbar]	Measured Bias [mbar]
6436.3	25.7452	25.7 ± 0.5	0.0 ± 0.5
6129.8	24.5192	24.6 ± 0.5	0.0 ± 0.5
5810.0	23.2400	23.1 ± 0.6	−0.2 ± 0.6
5287.2	21.1488	20.5 ± 0.6	−0.7 ± 0.6
3952.0	15.8080	15.8 ± 0.5	0.0 ± 0.6
3314.8	13.2592	13.1 ± 0.7	−0.1 ± 0.7
2797.8	11.1912	11.0 ± 0.7	−0.2 ± 0.7
2481.0	9.9240	9.7 ± 0.7	−0.2 ± 0.7
2064.2	8.2568	8.2 ± 0.5	−0.1 ± 0.5
1802.2	7.2088	6.7 ± 0.7	−0.5 ± 0.7
1487.0	5.9480	5.7 ± 0.6	−0.2 ± 0.6
1187.2	4.7488	4.6 ± 0.5	−0.1 ± 0.5
730.8	2.9232	2.9 ± 0.6	−0.1 ± 0.6
492.0	1.9680	1.8 ± 0.4	−0.2 ± 0.4
238.3	0.9532	1.0 ± 0.5	0.0 ± 0.5

**Table 2:** Results of the test for the nitrous oxide detection.

Total Pressure [mbar]	Partial Pressure [mbar]	Measured Partial Pressure [mbar]	Measured Bias [mbar]
1035.9	51.280	51.28 ± 0.10	0.00 ± 0.10
604.6	29.930	29.84 ± 0.30	−0.09 ± 0.30
293.8	14.547	14.49 ± 0.10	−0.06 ± 0.10
100.7	4.985	4.98 ± 0.09	0.00 ± 0.09
48.5	2.403	2.41 ± 0.07	0.01 ± 0.07
13.5	0.666	0.68 ± 0.09	0.01 ± 0.09
9.7	0.480	0.48 ± 0.06	0.00 ± 0.06
4.8	0.236	0.23 ± 0.08	0.00 ± 0.08

**Table 3:** Overview of the typical performance of other industrial combustion diagnostic techniques.

Technique		GC	Electrochemical	FTIR
Response time/sampling rate		Discrete sampling	T_90_ < 40 s (0–10 ppm)	5 Hz
Operational Range	NO	\	0–5000 ppm	0–2500 ppm
	N_2_O	0.1–100 ppm	0–20 ppm	0–100 ppm
	NO_2_	\	0–200 ppm	0–100 ppm
Limit of Detection	NO	\	5 ppm	0.2 ppm
	N_2_O	0.02 ppm	10 ppm	0.1 ppm
	NO_2_	\	0.5 ppm	0.2 ppm
Reference		[48]	[49,50,51]	[52]
Advantages		High sensitivity and accuracy, reference method, multi-species analysis.	Low cost, easy to install, low power consumption, simple electronic integration.	Multi-species detection, high accuracy, excellent linearity.
Disadvantages		Non real-time monitoring, complexity, use of gas carrier, maintenance, needs to be integrated with other instrument to have a complete overview over NO_2_ and NO_x_	Cross-sensitivity, limited lifespan, sensitivity to T/RH fluctuations, sensor-flow contact issues.	High cost, bulky setup, requires heated sampling lines to prevent water interference, saturation at high concentrations.

## Data Availability

The raw data supporting the conclusions of this article will be made available by the authors on request.
